# Interaction of Phytohormones and External Environmental Factors in the Regulation of the Bud Dormancy in Woody Plants

**DOI:** 10.3390/ijms242417200

**Published:** 2023-12-06

**Authors:** Zhaoyu Chen, Yadi Chen, Lanxi Shi, Li Wang, Weixing Li

**Affiliations:** College of Horticulture and Landscape Architecture, Yangzhou University, Yangzhou 225009, China; mz120221399@stu.yzu.edu.cn (Z.C.); yadichen@yzu.edu.cn (Y.C.); mz120231502@stu.yzu.edu.cn (L.S.); liwang@yzu.edu.cn (L.W.)

**Keywords:** bud dormancy, phytohormone, light, temperature

## Abstract

Bud dormancy and release are essential phenomena that greatly assist in adapting to adverse growing conditions and promoting the holistic growth and development of perennial plants. The dormancy and release process of buds in temperate perennial trees involves complex interactions between physiological and biochemical processes influenced by various environmental factors, representing a meticulously orchestrated life cycle. In this review, we summarize the role of phytohormones and their crosstalk in the establishment and release of bud dormancy. External environmental factors, such as light and temperature, play a crucial role in regulating bud germination. We also highlight the mechanisms of how light and temperature are involved in the regulation of bud dormancy by modulating phytohormones. Moreover, the role of nutrient factors, including sugar, in regulating bud dormancy is also discussed. This review provides a foundation for enhancing our understanding of plant growth and development patterns, fostering agricultural production, and exploring plant adaptive responses to adversity.

## 1. Introduction

Branches develop from axillary buds. Plant branching is a fundamental aspect of plant growth that not only determines plant architecture but also influences the cultivation and yield of crops and horticultural plants. Axillary meristematic tissue is formed in the leaf axils and develops into a bud primordium. The bud primordium absorbs nutrients and grows into new branches or remains dormant.

Dormancy is the temporary suspension of visible growth of any meristematic plant structure, and it occurs during the annual growth cycle of most temperate woody plants, including *Pinus*, *Cupressaceae*, and dicotyledons [1,2,3]. Bud dormancy manifests in both terminal and lateral buds of plants, triggered by the synergistic effects of shortened daylight hours and lower temperatures in late autumn, as an adaptive response to prepare for the impending cold winter season [4,5,6]. During evolution, perennial plants precisely balance the transition between the entrance and release of bud dormancy through gene regulatory mechanisms in response to environmental changes [7,8]. Analysis of transcriptome data from grape (*Vitis vinifera*), poplar (*Populus* L.), and tea tree revealed a close relationship between axillary bud dormancy and dormancy release processes with phytohormones and sugars, and they function as a complex network [9,10,11,12]. In addition, bud dormancy involves a complex physiological and biochemical process that is regulated by various environmental factors, such as light, temperature, and moisture.

## 2. Key Development Stages and Regulations of Bud Annual Growth Cycle

**Figure 1:**
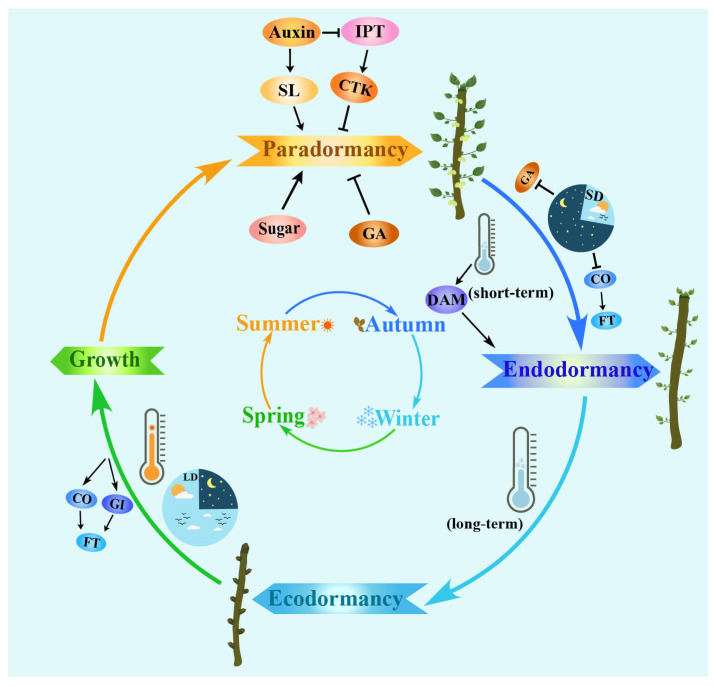
Model of the bud seasonal growth patterns of perennial trees in temperate regions. In temperate regions, the annual growth of phenological characteristics holds paramount importance for the germination of buds, development of shoot architecture, and timing of flowering in perennial plants [7,10,17]. There are four stages of bud dormancy and release: (1) growth cessation and bud set in early autumn, (2) establishment of bud dormancy in late autumn, (3) release of bud dormancy in spring, and (4) bud burst and active growth in spring and summer.

As there are no discernible physiological characteristics observed during each stage of plant dormancy, the definition of dormant stages is not very clear. Amen (1968) divided the process of bud dormancy in plants into four stages: induction, maintenance, triggering, and germination [13]. Lang (1987) proposed the three types of dormancy, including paradormancy, endodormancy, and ecodormancy, as the most widely accepted classification based on their causes [14]. Paradormancy, which is also referred to as relative dormancy, is caused by external factors like leaves or apical dominance that affect buds [15]. The buds remain incapable of initiating sprouting and generating fresh branches or foliage. However, if the adjacent tissue is removed, the dormant buds can resume growth quickly [16,17]. Significant progress has been made in studying the complexities of paradormancy, especially in understanding the mechanisms behind apical dominance [18,19,20]. Endodormancy is controlled by the various factors inherent within the dormant buds, including the chilling requirement and photoperiod [21,22,23]. Despite favorable external environmental conditions or without adjacent organ limiting factors, the dormant buds will not germinate unless they undergo specific treatments, such as chilling for a certain time [24,25]. Hence, ecodormancy is also known as spontaneous dormancy, induced dormancy, or natural dormancy. When severe environmental adversity is encountered, such as extreme temperature fluctuations, water logging, drought stress, or nutrient deficiencies, buds cease growth and enter an ecodormancy state. Ecodormancy will be released, and bud break typically occurs when unfavorable environmental conditions are removed [8,26]. In the annual cycle of woody plants, the three types of dormancy mentioned above are interrelated and dynamic processes wherein bud states can transition from one to another [Figure 1]. In other words, buds develop during the growing season (spring–autumn) and remain in a dormant state until the reproductive bud burst occurs in the next spring.

The process of bud outgrowth can be categorized into three distinct phases: rapid bud activation, germination, and continuous growth. The transmission of these signals is often facilitated by environmental factors and phytohormones, particularly auxin, cytokinin (CTK), strigolactone (SL), and gibberellin, which play crucial roles in coordinating and regulating the growth of axillary buds [27,28]. These factors have a relationship of mutual interaction. The main environmental factors—light and temperature signals—are located either upstream or in the most central position. Bud development is regulated by hormone signals that are mediated by light [29,30,31].

## 3. Hormonal Regulation of Bud Activation and Outgrowth

The regulation of bud outgrowth is a complex process that involves intricate signaling pathways mediated by phytohormones. These phytohormones interact and influence each other in diverse and dynamic ways, which adds a layer of intricacy to the overall regulatory mechanisms [32]. 

### 3.1. Auxin Regulation of Bud Outgrowth

Apical dominance refers to a phenomenon where the growth of axillary buds is inhibited by the main stem in some plant species. Apically dominant plants channel their energy towards the apical meristem, which is located at the tip of a shoot axis [33]. However, in the case of damage or the absence of the apical meristem, the dormant axillary buds become stimulated [34]. Auxin, widely investigated for more than a century, stands as the pioneering hormone in scientific research. It is well-established that auxin plays a crucial role in the progress of plant meristem development [35,36]. Axillary buds have the potential to break dormancy and grow into lateral branches when the apex is decapitated, and the application of auxin to the top of decapitated seedlings will re-inhibit branching [37,38,39]. In contrast, the exogenous application of auxin transport inhibitors (N-1-naphthylphtha-lamicacid, NPA) to intact stems eliminates apical dominance and induces axillary bud outgrowth [40].

Auxin is primarily synthesized in the actively growing tip of plants, which includes the stem apex and young leaves. It is then transported from the tip to the base through the polar transport stream (PATS) in order to sustain apical dominance [37,41,42]. Additionally, it is hypothesized that the inhibitory effect of auxin on axillary buds is not a direct effect, but instead, it is mediated by a second messenger. It is posited that auxin engages in interactions with downstream second messengers, such as CTK and SL, to indirectly govern the process of axillary bud germination [43,44]. Although it is firmly established that auxin inhibits bud outgrowth through downward polar transport, the mechanism is still not completely understood. In perennial woody plants, the removal of terminal buds triggers the release of apical dominance, resulting in the sprouting of lateral buds. Similarly, *Rosaceae* fruit trees showcase the sprouting and flowering of various types of buds, such as lateral, terminal, flower, and leaf buds, following the autumn leaf fall. It is noteworthy that early defoliation promotes floral bud germination by causing the polar distribution of the auxin efflux carrier protein PpyPIN1b on the cell membrane in pear (*Pyrus pyrifolia*) [45]. Early defoliation induces germination not only in lateral buds but also in terminal buds. This finding suggests that bud dormancy during this period is not significantly influenced by terminal dominance but is dependent on the presence or absence of leaves, suggesting that compounds or signals from the leaves may regulate paradormancy [45]. Mature leaves possess the ability to synthesize growth hormones, which are subsequently transported to buds, effectively suppressing shoot germination [46]. Moreover, the removal of leaves triggers the redistribution of auxin within buds and their basal stem segments, facilitating the release of bud paradormancy. Additionally, CTK and SL also play an important role in this process, although their mechanisms of action remain unclear. 

### 3.2. The Crosstalk of Auxin and CTK in Bud Germination

CTKwas initially recognized to play a role in cell division and the development of adventitious buds. They are predominantly produced in the root tip and are subsequently transported to other areas of the root and aerial parts of the plant [47,48]. However, it is worth noting that aerial parts, such as leaves and stem nodes, can also synthesize CTK [49]. CTK has been recognized as the primary phytohormones that are responsible for directly promoting lateral bud outgrowth as the second messenger of auxin, and they perform antagonistic functions in bud outgrowth [50].

In the stem, high levels of auxins inhibit the transport of CTK to the shoots. The external application or inducible biosynthesis of CTK can promote the synthesis and transport of auxins as well as bud outgrowth [51]. This indicates that auxin and CTK have both synergistic and antagonistic effects on axillary bud growth. In *Zanthoxylum bungeanum*, 3-year-old transgenic plants overexpressing *CaIPT* displayed the typical morphology of CTK overexpression, such as reduced stem elongation, increased leaf area, and more branches [52]. The ectopic expression of the apple *MdIPT3a* gene in tobacco promoted axillary bud germination [53]. In apple, *MdIPT1* was highly expressed in axillary buds and induced axillary bud germination. The overexpression of *MdIPT1* in *Arabidopsis* resulted in multi-branching [54]. The mechanisms were conserved in annual plants. For example, in *Arabidopsis*, *ipt* and *arr* multiple mutants exhibited fewer branches compared to wild-type plants. However, the response of these mutants to auxin in the isolated nodal segment bud (without root-derived CTK) was not impaired as compared to the wild type [55]. CTK signaling is also involved in the regulation of axillary meristem formation. Severe defects in axillary meristem initiation occur because of mutations in B-type ARR transcriptional factors, which play a role in CTK signaling. In *Arabidopsis*, B-type ARRs can bind to the *WUS* promoter in order to activate and stabilize *WUS*. This helps to restrict its signaling in the central zone of the shoot apical meristem [56,57]. 

Auxin and CTK have opposing roles in regulating axillary bud germination; they are intrinsically regulated by each other [7,57,58,59]. Root-derived CTK has been attributed to the antagonistic effects of auxin in the bud outgrowth response to decapitation, and the content of CTK in the xylem-sap increases after decapitation and accumulates in buds in many species, such as apple [60]. The decrease in auxin levels leads to the release of inhibition of *IPT* expression, which promotes the outgrowth of axillary buds through local CTK synthesis, particularly the ZR CTK pool after decapitation [61]. In *Arabidopsis*, the inhibition of CTK synthesis by auxin is rapid and dependent on the *AXR1* gene-mediated auxin signaling pathway. Similarly, in pea, auxin can decrease CTK levels by down-regulating the expression of *IPTs* and up-regulating the degradation gene *CKX2* [62]. On the other hand, CTK exerts a regulatory effect on the polar transport of auxin. The inhibitory effect of auxin on axillary bud activity can be counteracted by applying exogenous CTK. In addition, CTK regulates the accumulation of transcripts of auxin efflux transporters *PIN3*, *PIN4*, and *PIN7*, thereby promoting shoot branching in the *arr1* mutant [63,64,65]. Moreover, the application of CTK also induces an increase in the auxin content in the young roots of plants [66]. These findings indicate that CTK plays a dynamic role in regulating the distribution and level of auxin in response to changes in the external environment. However, the increased content of CTK and bud outgrowth can be prevented by providing exogenous auxin [67,68,69]. 

In summary, CTK promotes branching by activating axillary buds. However, the mechanism is not well understood. In *Arabidopsis*, the *arr 3/4/5/6/7/15* mutants lacking the A-type ARR proteins reduced branching compared to the wild type, which is contrary to the A-type ARR acting as a negative regulator of CTK signaling [67,70]. The *arr1* mutant responds to CTK signaling and increases branching, which is consistent with the antagonistic effect of type A-type ARRs but clearly contradicts the previously reported positive role of CTK in bud activation [67]. Therefore, CTK may also regulate branching through other mechanisms. 

### 3.3. The Function of Strigolactone in Bud Outgrowth

SL is synthesized in roots and shoots to promote the growth of lateral roots and inhibit the growth of lateral branches, respectively [71]. Several species, including pea, apple, petunia, and *Arabidopsis*, have shown evidence of a negative regulatory role of SL in vegetative meristems. The functional mutants of the SL signal pathway often present more branches [72,73]. In most species, such as peas, rice, and *Arabidopsis*, SL synthesized in the roots plays a crucial role in inhibiting axillary bud germination. However, in apple, axillary bud sprouting is controlled by SL synthesis in branches but not by SL produced in roots [74]. Two hypotheses have been proposed from numerous studies on plant meristem development. Firstly, SL acts as a second messenger, influencing axillary bud germination by regulating the transport of auxin in the apical meristem. Secondly, for most plants, SL functions as a long-range signaling substance, directly inhibiting axillary bud germination through bottom–up transport into plant buds [75]. 

Multiple studies have demonstrated feedback regulation between SL, auxin, and CTK in the context of plant meristem development. Auxin plays a role in regulating the synthesis of SL in plant stems [29,76,77]. This suggests that SL can act downstream of auxin to enhance the inhibiting effect of the outgrowth of axillary buds. This direct action is similar to the CTK, which is regulated by the AXR1-AFB signaling pathway. The apical production of auxin controls the expression of SL synthesis genes in *Arabidopsis*. When the auxin source is removed through decapitation or deletion, the SL synthesis gene in the stem is down-regulated, leading to the inhibition of SL synthesis [29]. However, SL is still considered a secondary signaling molecule for auxin. The second messenger hypothesis for auxin proposes that SL inhibits axillary bud germination by regulating the expression of the TCP gene family (*BRANCHED 1*, *BRC1*) and suppressing cell cycle and meristematic tissue activity in axillary buds. SL can induce the expression of the rice *OsCKX9* gene, which is responsible for CTK degradation [78]. This leads to a down-regulation of CTK levels, subsequently triggering the response of downstream genes. Both SL and CTK play a direct role in regulating lateral bud development, and it is possible that they both regulate a shared component, the *BRC1* gene [79]. 

### 3.4. Other Hormones

Gibberellic acid (GA) plays a key role in dormancy release in deciduous fruit trees. It is primarily synthesized in the young apical parts of plants, such as stem and root tips, as well as in developing seeds and fruits. Although mature leaves can synthesize GA, it is usually not transported to other parts of the plant. GA synthesized at the root tip can be transported upwards through the xylem ducts. Similarly, the GA produced in the terminal buds and young leaves can be transported downwards through the sieve tubes in the phloem. GA has been found to play a crucial role in both the establishment and release of endodormancy induced by low temperatures and short days [2,80]. Several studies have demonstrated that the application of exogenous GA sprays promotes the release of dormancy effectively in deciduous fruit trees. This may be attributed to the capacity of GA to partially substitute the requirement for cold temperatures, thus facilitating the release of dormancy [81,82,83]. Poplars with lower levels of active GA exhibit delayed dormancy release [84]. Furthermore, the external application of GA_4_ was observed to enhance the expression of the GA metabolism genes *GA20ox* and *GA2ox* [85]. These genes work in synergy with Ca^2+^ and reactive oxygen species (ROS) signals to facilitate the release of natural dormancy and promote bud germination [86,87].

Researchers have identified several physiological functions of GA in the release of dormancy in plants: (1) The primary physiological function of GA is to promote plant growth, specifically cell elongation. Studies have demonstrated that treating dwarf plants with GA has a positive impact on growth, specifically focusing on promoting internode elongation [88]. Unlike auxin, GA promotes plant growth without concentration dependence. (2) GA can contribute to the energy required for normal plant life activities. It achieves this by stimulating hydrolytic enzymes like proteases, which degrade stored materials and provide energy. Therefore, GA plays a role in breaking plant dormancy to some extent, instead of light and temperature [85]. (3) Flowering bud differentiation in higher plants is typically regulated by photoperiod and temperature [88]. GA can induce flowering in long-day plants as well as influence flowering in plants that have already completed flower bud differentiation [89].

The release of bud dormancy by GA is a complex regulatory mechanism that depends on the dormancy status of the bud, as well as the species and concentration. Increasing evidence has shown that, under natural conditions, a sustained period of low temperature is the only way to induce the release of physiological dormancy. In poplar, a long-term low-temperature treatment induced the expression of GA biosynthesis genes. Additionally, during dormancy, the external application of GA stimulated the expression of the β-1,3-glucanase gene (*GH17*), which could degrade the 1,3-β-glucan (callose) in plasmodesmata, inducing the opening of the symbiosis pathway in the apical meristem (including symplasm, cell walls, or both), the increase of intercellular filaments, the enlargement of channel apertures, and the gradual restoration of intercellular communication, resulting in the release of dormancy [90,91]. In poplar, after five days of GA_4_ feeding, the sphincters that obstruct PD during dormancy had disappeared [91]. In response to low temperatures, the expression of genes related to GA synthesis was up-regulated in apical meristem cells [91], and large quantities of GA were synthesized. Increased GA content promotes the degradation of callose in the sieve tubes, enabling the long-distance transport of *FT* and *GH17*, resulting in the release of dormancy. When the temperature increases, FT moves to the apical meristem and then completes the process of bud sprout and elongation later [91].

The inhibition of bud growth by abscisic acid (ABA) has been hypothesized for a long time because of the observation that the supply of exogenous ABA inhibits bud outgrowth [19,92]. During dormancy, the ABA content increases gradually and reaches peak content when entering deep dormancy [93,94]. The levels of ABA in the buds gradually decrease as the buds transition from the dormancy period to germination [95,96,97]. When the defoliation of plants is delayed and their ABA content increases, the dormancy of plants deepens, requiring a longer accumulation of low temperatures to break plant dormancy. Consequently, there is a negative correlation between bud germination and the ABA content [92,98,99].

In grape buds, ABA levels increase from paradormancy to endodormancy and decrease during endodormancy release. During peach bud dormancy periods, ABA levels are high during endodormancy and reach their lowest levels during ecodormancy. Tuan et al. (2017) also found that in ‘Kosui’ pear, ABA levels increase during endodormancy, followed by a decrease during the transition to ecodormancy [100]. All of these findings indicate that ABA plays a role in regulating dormancy processes in perennial plants. The ABA content in buds increases during the onset of natural dormancy in late autumn and decreases rapidly after exposure to low temperatures or dormancy-inducing treatments. This rapid decrease in ABA content is accompanied by changes in the expression of genes involved in ABA biosynthesis and degradation [95,97]. For example, during the dormancy induction period and dormancy, the expression of the ABA biosynthesis genes encoding *NCED* (9-cis-epoxycarotenoid dioxygenases) is up-regulated [97].

While ABA does play a part in regulating the endodormancy process of shoots, it is important to note that other factors are also involved in causing plants to enter dormancy [101]. Bud dormancy is a complex process that is influenced by multiple hormones. It was found that the application of ABA on grapevines at 100 mg/L or 1000 mg/L did not delay bud dormancy in spring [102]. This suggests that a number of other variables, in addition to ABA, are likely at play in the regulation of dormancy.

### 3.5. How Sugar Interacts with Hormones

Light-regulated sugar, a vital source of energy and carbon, contributes to the development of cell wall components necessary for bud outgrowth. Shoot decapitation removes the sink for sugar and induces long-distance transport, resulting in rapid sugar distribution and accumulation in the buds, initiating rapid basal bud outgrowth [103]. A strong correlation has been observed between sugar availability and bud outgrowth in cases of defoliation, which leads to a disruption in the source–sink relationships for sugars [104,105].

From a growth perspective, axillary buds are considered sink organs that are less photosynthetically active [106]. They rely on importing sugars to meet their metabolic demand and support their growth. The growth capacity of the bud is determined by its ability to acquire and utilize sugars. As a result, buds engage in competition for sugars in order to sustain their growth. Sugars may be the early signals of bud outgrowth after decapitation [104]. When the shoot tips of pea are removed, the sugar is redistributed and accumulates in the basal buds quickly before the auxin contents change. A strong correlation between the supply of sucrose and the germination of axillary buds has also been found in sorghum, pea, and wheat [104,107,108]. Applying sucrose analogs to pea, *Arabidopsis*, or rose stem segments in vitro can effectively activate axillary bud germination. Except for defoliation, all other factors that affect sugar content, such as increased CO_2_ supply [109,110] and the inhibition of sucrose degradation, may impact the outgrowth of buds [111].

In the regulation of bud outgrowth, sugars are proposed as signals, and this progress is partially controlled by trehalose 6-phosphate (Tre6P), which indicates the sugar levels. Plants overexpressing *AtTPS* (the major Tre6P biosynthetic gene) exhibit higher levels of Tre6P content and an increased number of branches. Consistently, the plants overexpressing *AtTPP* (the Tre6P major degradative gene) showed a significant reduction in both Tre6P content and the number of branches. In maize, the *ramosa3* (*ra3*) mutant exhibits increased branch formation due to the mutation of *trehalose-6-phosphate phosphatase* (*TPP*) [112]. Additionally, decapitation rapidly alters the expression of genes associated with sugar transport, metabolism, and signaling. This observation further supports the idea that the growing shoot tips inhibit bud outgrowth by depriving the axillary bud of sugars [104,113,114,115].

Sugar interplays with hormones to regulate plant growth and development. Previous studies have confirmed that sucrose can enhance the production of CTK in rose nodes when cultured in a growth medium. But, in fact, replacing sucrose with CTK is not enough to trigger bud outgrowth. Feeding sucrose to detached potato stems under etiolated conditions stimulates the CTK accumulation directly or indirectly prior to the initiation of stem branching [116].

During bud outgrowth, an antagonistic relationship exists between sugar and auxin. In other words, sugar acts prior to auxin in order to alleviate its inhibitory effects on bud outgrowth. While auxin can also impact sugar metabolism and transport, its main function is to restrict starch breakdown and regulate the accumulation of soluble sugars, like sucrose, in fruits. It controls the distribution of sugars between sources and sink organs. This process is mediated by sugar transport proteins, such as sugar transporters (SUTs). Recent studies indicate that in pear trees experiencing early defoliation, the regulation of sugars for bud dormancy may extend beyond their transport and metabolic pathways [45]. It is suggested that the combined action of signal transduction pathways also plays a role in this process.

### 3.6. Hormones Respond to External Environmental Factors

**Figure 2:**
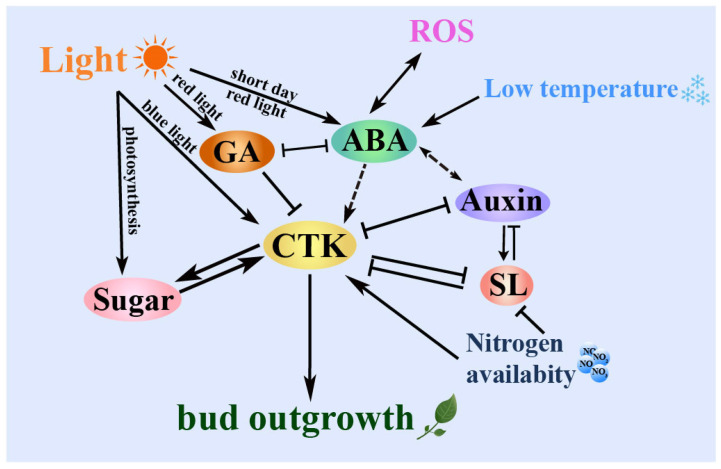
The process of bud germination is indirectly influenced by external environmental factors and nutritional elements. These factors and elements regulate hormone synthesis and decomposition metabolism, ultimately resulting in bud germination induced by CTK. Light, sugar, and nitrogen directly stimulate CTK synthesis, leading to the release of bud dormancy. Low temperature and ROS signals indirectly trigger CTK synthesis by up-regulating ABA signaling, ultimately promoting bud germination. Arrowheads indicate positive regulation, flat-ended lines denote negative regulation, and bilateral flat lines represent mutual inhibition. Solid lines indicate direct regulation, while dashed lines indicate indirect regulation.

Environmental factors, such as light and temperature, exert a substantial impact on the intricate processes of bud outgrowth and development. Light is not only the primary source of energy for plants but also an essential factor that influences their growth and development. Plants detect variations in the external light surroundings through photoreceptors, which include phytochromes (PHY), blue light receptors, and UV-B receptors. They utilize this information to adapt their structure and control processes such as bud dormancy and germination. Numerous studies have revealed that light signals interact with phytohormones, including auxin, GA, CTK, ethylene (ETH), and brassinosteroids (BR), through three key aspects: light intensity, light quality, and photoperiod, significantly impacting biosynthesis and signaling processes [Figure 2].

Plants in natural environments must adapt to survive in reduced light intensity due to shading from nearby vegetation. A previous study has shown that applying continuous shading treatment during bud dormancy significantly hinders bud break in litchi plants. [117,118]. Seasonal changes in light quality can indirectly impact the induction and release of bud dormancy in plants by regulating physiological metabolic processes. Phytochrome-interacting factors (PIFs) play a crucial role not only as pivotal regulators in the light signaling pathway, where they receive light signals from red and blue light receptors PHY and CRY (Cryptochrome), but also in the regulation of bud outgrowth by controlling other hormonal pathways. Changes in light intensity can regulate plant branching by affecting the levels of the branching-promoting second messenger CTK. The low red/far red ratio can induce CTK degradation, thereby inhibiting wheat tillers [119]. In *Arabidopsis* seedlings, PIFs suppress the synthesis of GA through the modulation of DELLA proteins and promote the synthesis of ABA, two processes that are believed to inhibit bud outgrowth.

The photoperiod refers to the cyclical changes in the length of light and dark periods during a day–night cycle, varies with the changing seasons, and serves as a key environmental factor regulating bud dormancy and germination, particularly in deciduous tree species. Short days induce dormancy in many tree species, whereas long days stimulate bud development [120,121,122]. When the duration of daylight is less than 10–12 h, the branches of *Norway maple*, poplar, and spruce halt their growth, and buds enter a dormant state [120]. The *CO* gene (*CONSTANS*) plays a crucial role in the photoperiod pathway and serves as a key component in regulating the biological clock and flowering time genes. It triggers the expression of the downstream gene *FLOWERING LOCUS T* (*FT*), which induces bud development. On the other hand, short days inhibit the expression of *FT*, thereby suppressing bud development [123]. Other clock genes like *SOC1* and *LHY* also have significant regulatory functions in bud development in trees [124]. Notably, the expression levels of the clock genes *GIGANTEA* (*GI*), *FLAVIN BINDING KELCH REPEAT F-BOX1* (*FKF1*), and *EARLY FLOWERING4* (*ELF4*) increase significantly during the transition from ecodormancy to endodormancy [125,126]. In addition, genes involved in hormone synthesis and metabolism, such as CTK, auxin, ABA, and GA, demonstrate rhythmic behavior in various plant species. The synthesis and signaling pathways of plant hormones, such as CTK and auxin, can be regulated by the photoperiod and play a role in controlling the dormancy and germination of buds [127,128]. In poplar, short days induce the production of ABA, leading to increased expression of *ABA-INSENSITIVE3* (*ABI3*). *ABI3* inhibits the formation of closed apical buds induced by short days, promoting bud development while suppressing plant growth [129]. Short days can also promote dormancy in *Picea asperata* and induce late activation of genes related to dormancy and freezing tolerance [130]. The aforementioned research implies that light can regulate hormone synthesis, metabolism, and gene expression involved in the bud development process through light quality, light intensity, and photoperiod.

Aside from light signals, temperature fluctuations synchronize with the changing seasons. Certain plants, such as species in the *Rosaceae* family, like pear and apple, require low temperatures in order to undergo dormancy. The release of endodormancy in deciduous fruit trees necessitates a specific period during winter known as chilling requirement (CR), and the required chilling hours vary among different species. The MADS-box gene *SVL* (*SHORT VEGETATIVE PHASE LIKE*) is triggered by ABA, emerging as a crucial transcription factor, orchestrating the induction of endodormancy [91,131]. The MADS-box proteins that are associated with dormancy are referred to as DAMs in *Rosaceae* plants. The ectopic expression of *PmDAM6* in poplar causes the formation of dormant terminal buds, suppressing growth, while in apple, the overexpression of *MdDAMb* and *MdSVPa* results in a delayed bud outgrowth [132,133]. Short-term exposure to low temperatures increases the ABA content within pear buds, thereby activating the expression of *ABRE-BINDING FACTOR3* (*PpyABF3*). ABF3, by directly or indirectly activating the expression of *CBF4*, leads to the expression of *DAM3* to maintain bud dormancy [134]. Long-term exposure to low temperatures dynamically induces epigenetic changes in poplar, leading to altered gene expression (including *DAM* and *CYP707A*), which in turn affects the levels of ABA and ultimately triggers the release of endodormancy [2]. In summary, external temperature changes can also lead to the accumulation or decrease of ABA, thereby promoting or inhibiting the growth and development process of buds.

### 3.7. Hormones Respond to Nutritional Factors

The process of bud outgrowth requires a significant number of nutrients, making nutritional factors crucial. Specifically, certain essential nutrients, such as nitrogen and phosphorus, have the ability to influence bud germination by intricately modulating the dynamic changes in hormone levels. Nitrogen may exert control over branching through modulation of a second messenger, thereby influencing the levels of CTK and SL. Another study found that inadequate nitrogen levels in the soil significantly decrease the content of CTK in wheat, suggesting that nitrogen plays a crucial role in determining CTK synthesis [135]. Further studies have found that rice tillering number increases with nitrogen application, which may be caused by increased CTK synthesis due to increased nitrogen levels [127,136]. In *Phyllostachys*, the lack of phosphorus inhibits tillering bud development and re-tillers, and phosphorus relies on *REV* and *TB1* genes as well as the expression of auxin, CTK, SL synthesis, and transporter genes to mediate tillering bud development and re-tillers [137].

Other signaling molecules, such as ROS, also act as regulators in the process of hormone signaling to induce plant dormancy, growth, and development, intricately participating in the precise orchestration of plant dormancy, growth, and development. Remarkably, ROS acts as a stimulant for regulating the dormancy period of buds by releasing informative signals, thereby orchestrating notable alterations in carbohydrate levels and hormone metabolism within the bud [Figure 2]. This interplay endows the bud with robust resilience against cold stress, effectively facilitating its progression toward endodormancy. Previous investigations involving *Arabidopsis* catalase (*cat2-1*) and vitamin E (*vet1-1*) seeds have concisely demonstrated that the presence of ROS effectively initiates the germination process by intricately activating GA signaling pathways [138]. These complicated interactions occur through unique molecular mechanisms within plants, exerting a pivotal influence on their developmental programs and adaptive responses to the environment.

## 4. Conclusions and Perspective

In recent years, significant progress has been made in understanding bud dormancy and germination, particularly in relation to the role of plant hormones. Among these hormones, auxin plays a crucial role as a key regulator of growth and development during bud dormancy and germination [34,61]. Similarly, CTK promotes cell proliferation and division, facilitating the germination process [47,48]. The roles of GA and SL in regulating bud dormancy and germination have also been extensively studied [71,72,73,81,82,83]. Additionally, the role of sugar signaling in this process cannot be underestimated, as it interacts with hormone signaling pathways to regulate gene expression, metabolic control, and hormone signal transduction. Moreover, external environmental factors also play a crucial role in the intricate process of bud dormancy and germination. Various elements, including light, temperature, and a variety of factors, intricately interweave and coordinate the modulation of signaling pathways that involve plant hormones [43,92,120,121,130].

To summarize, the molecular complexities that govern bud dormancy and germination constitute a delicate and intricate regulatory network. This network is intricately woven through the collaborative interplay of plant hormones, sugar signaling, and external environmental factors [20,86,87,104,113,138]. Investigating these profound molecular mechanisms in greater depth holds immense significance, as it provides us with a deeper understanding of the fundamental patterns of plant growth and development. This knowledge empowers the advancement of agricultural production and helps in navigating unexplored territories of plant adaptability in the face of challenges. For example, currently, global warming is causing bud to sprout after leaf fall. This phenomenon adversely affects fruit trees, particularly those belonging to the *Rosaceae* family, which are typically cultivated in temperate regions. As a consequence, these fruit trees produce fewer fruits in the second-year spring season [45]. Additionally, excessive fall flowering has a detrimental impact on the number of flowers produced during the following spring, subsequently leading to decreased yields. Moreover, global warming may reduce the CR of plants, resulting in delayed flowering, prolonged flowering periods, or even an outright failure to bloom. In future research, it is necessary to strengthen our exploration and gradually uncover the mysteries of these complex interactions.

In fruit tree production, specifically in *Rosaceae* fruit trees, there is a phenomenon where the trees may flower again in the fall of the same year after defoliation. This abnormal physiological occurrence not only depletes the tree’s nutrient storage, leading to a deficiency in the tree buds for the following year, but also results in the loss of fruit buds. Consequently, this leads to reduced flowering and fruit yield in the subsequent year. Furthermore, trees such as *Ginkgo biloba*, which primarily collect leaves, have the ability to significantly increase their yield and economic efficiency by harvesting leaves multiple times a year [139]. This highlights the importance of axillary buds sprouting multiple times throughout the year. Therefore, further research is needed to explore how hormones and key genes integrate to regulate the molecular mechanisms involved in the release of bud dormancy after defoliation, which is different from apical dominance.

With the advancement of new analytical tools and techniques, future studies on phytohormones will focus on enhancing our understanding of the physiological functions, transport mechanisms, signal transductions, and hormone interactions mediated by environmental signals. The following four aspects will be particularly strengthened: (1) identifying novel phytohormones that regulate bud dormancy and outgrowth; (2) investigating the mechanisms underlying the synergistic or antagonistic effects of multiple phytohormones in regulating bud dormancy and outgrowth; (3) examining the impact of external environmental factors, such as light and temperature, on phytohormone regulation of bud dormancy and outgrowth and exploring the role of epigenetic regulatory mechanisms, such as DNA methylation and miRNA shearing, in influencing bud dormancy and outgrowth; (4) aiming to achieve directed regulation of bud dormancy and outgrowth through exogenous phytohormone application to enhance plant productivity. In conclusion, further comprehensive research is necessary to fully comprehend the intricate and complex mechanisms through which phytohormones regulate bud dormancy and break, considering the influence of environmental factors.
